# Expression of some circulating microRNAs as predictive biomarkers for prognosis and treatment response in glioblastoma

**DOI:** 10.1038/s41598-024-83800-x

**Published:** 2025-01-14

**Authors:** Elham Ali, Marwa Adel Ahmed, May A. Shawki, Lobna R. Ezz El Arab, Mohamed K. Khalifa, Menha Swellam

**Affiliations:** 1 Molecular Biology, Zoology and Entomology Department, Faculty of Science (for Girls), Al-Azhar University, Nasr City, Cairo, 11754 Egypt; 2 Clinical Pharmacy Department, Faculty of Pharmacy, Ain Shams University, Cairo, Egypt; 3 Clinical Oncology Department, Faculty of Medicine, Ain Shams University, Cairo, Egypt; 4 CSO at Omicsense, Cairo, Egypt; 5 Molecular Pathology Laboratory Children Cancer Hospital, Cairo, 57357 Egypt; 6 Biochemistry Department, Biotechnology Research Institute, National Research Centre, Dokki, Giza, Egypt; 7 High Throughput Molecular and Genetic Laboratory, Central Laboratories Network and the Centers of Excellence, National Research Centre, Dokki, Giza, Egypt

**Keywords:** Glioblastoma, miR-29a, miR-106a, miR-200a, Biomarkers, In silico analysis, Biochemistry, Cancer, Molecular biology

## Abstract

Glioblastoma multiforme (GBM) is the most prevalent, treatment-resistant, and fatal form of brain malignancy. It is characterized by genetic heterogeneity, and an infiltrative nature, and GBM treatment is highly challenging. Despite multimodal therapies, clinicians lack efficient prognostic and predictive markers. Therefore, new insights into GBM management are urgently needed to increase the chance of therapeutic success. Circulating miRNAs (miRs) are important regulators of cancer progression and are potentially useful for GBM diagnosis and treatment. This study investigated how miR-29a, miR-106a, and miR-200a affect the prognosis of GBM patients. This study was conducted on 25 GBM patients and 20 healthy volunteers as a control group. The expression levels of target miRs were analyzed pre- and post-treatment using qRT-PCR and evaluated in relation to both clinical GBM criteria and the patient’s survival modes. The diagnostic efficacy of target miRs was assessed using the receiver operating characteristic (ROC) curve. MiRs levels showed significant differences among the enrolled participants. All investigated miRs were significantly elevated in GBM patients with non-frontal lesions. Only miR-200a showed a significant difference in GBM patients older than 60 years with a tumor size ≥ 5 mm. Regarding miR-106a, a significant difference was detected based on the surgical strategy and use of an Eastern Cooperative Oncology Group (ECOG) performance status equal to 2. For miR-29a, a significant upregulation was detected according to the surgical strategy. All post-treatment miRs levels in GBM patients were significantly downregulated. In conclusion, circulating miRs revealed a significant role in predicting GBM patient treatment outcomes providing valuable insights for personalized therapeutic strategies.

## Introduction

Glioblastoma (GBM) is the most dangerous form of central nervous system (CNS) malignancy, arising from neuroepithelial glial cells. Due to its aggressive invasion, genetic heterogeneity, devastating malignancy, and high rate of proliferation, GBM has a very dismal prognosis^1^. Over the past two decades, the infiltrative growth and heterogeneity of GBM have made it difficult to pinpoint an effective therapeutic strategy under recurrence-free conditions^2^. Currently, most biomarkers available for GBM diagnosis and predicting treatment outcomes require a tumor tissue biopsy, which has limitations due to risk-related procedures^3,4^. Furthermore, it is difficult to gather longitudinal samples to monitor therapeutic efficacy and detect intratumoral heterogeneity in GBM^4^. Additionally, the traditional methods used for the diagnosis and surveillance of GBM, such as computed tomography (CT) and magnetic resonance imaging (MRI), are very expensive, and CT carries the risk of radiation exposure^5^. The introduction of Isocitrate dehydrogenase (IDH) status to WHO CNS5 classification in 2021 reflects its importance in diagnosis and disease management^6^. In addition, O6-methylguanine-DNA methyltransferase (MGMT) promoter methylation predicts the patient’s response to chemotherapy, where they exhibit better outcomes with temozolomide (TMZ)^4^. To date, no molecular biomarkers in biological fluids can be used for identifying, monitoring, and predicting GBM. Therefore, there is a critical need for a new, less invasive way to assess the molecular biology of GBM to aid in its monitoring and management. Recently, circulating biomarkers such as miRNAs have gained attention as minimally invasive alternatives for GBM diagnosis and prognosis^3^.

MiRNAs (miRs) are short endogenous noncoding RNAs (~ 22 nucleotides) that have a major posttranscriptional regulatory impact on mRNA stability and translation, which in turn influences gene expression in multicellular organisms^7^. MiRNAs are substantial regulators of differentiation and development; they are dysregulated in all human malignancies and can act as oncogenes or tumor suppressors^8^. Dysregulation of miRs expression has been linked to the onset and progression of complex diseases, including cancer, cardiovascular diseases, immune response, and neurological disorders^9^. They can regulate signaling pathways involved in cellular processes, including cell differentiation, proliferation, and apoptosis^10^. The discovery of miRs revolutionized the diagnosis and prognosis of several diseases, including cancer^11–14^. They exist in biofluids, and recently, researchers have viewed them as an urgent tool for overcoming intrusive detection methods^11,15–17^ and treatment resistance^15,18^. Advances in computational models have enhanced the prediction of miRNA-disease associations, leveraging machine learning and data fusion techniques to integrate biological data, offering insights into miRNA functions and their potential as diagnostic or therapeutic targets^9,19,20^.

It is clinically important to identify new prognostic markers for predicting patient outcomes in GBM patients, which may shed light on the underlying biological processes driving the onset and progression of GBM^15,21^.

The microRNA-29 (miR-29) family has been reported to be correlated with the prognosis and aggressiveness of malignant tumors, so it may serve as an effective molecular indicator for identifying the beginning, development, and etiology of cancer. Three of the miR-29 family members, miR-29a, miR-29b, and miR-29c, have been found to be expressed in human malignancies. According to previous studies^22,23^, the three mature members are abnormally dysregulated in human cancer and influence their target genes through different cellular pathways, such as proliferation, migration, and apoptosis. In a prior study, it was reported that miR‐29a has an impact on protein kinase B (AKT) signaling, regulation, and suppression of the translation of the transcription factor Sox4, which causes growth activation and invasion of GBM^24^. Furthermore, miR-29a controls the platelet-derived growth factor (PDGF) pathway and targets TNF receptor-associated factor 4 (TRAF4)/Akt signaling, which inhibits the stemness and tumor formation of glioma cells^25^. MiR-29a can induce apoptosis through the modulation of P53 activity^26^. In addition, another study by Shi et al.^27^ on gliomas revealed that the miR-29 family enhances apoptosis in a p53-dependent manner through CDC24/PAK/AKT/MDM2 pathway suppression.

MiR-106a is a member of the miRNA-17 family, which is broadly conserved and involved in a variety of biological pathways. Mature miR-106a is ~ 23 nt in length and situated on Xq26.2^28^. A previous study demonstrated that upregulation of miR-106a-5p stimulates tumor cell proliferation and invasion in several types of solid tumors^29^; cumulative results showed that miR-106a could act as an oncomiR or tumor suppressor in a variety of malignancies, depending on the cellular context. Previous studies^28,30,31^ have revealed the oncogenic effect of miR-106a in GBM. Zhi and his colleagues^32^ reported that miR-106a acts as a tumor suppressor during astrocytoma development by targeting Fas-activated serine/threonine kinase (FASTK) in humans. Moreover, miR-106a increases p53 expression through E2F1 inhibition, while its effect on glioma cell proliferation is independent of p53 status^33^. In addition, Dai and his colleagues^34^ reported that regardless of p53 status, miR-106a suppresses the proliferation of human glioma cells and induces apoptosis by targeting E2F1. Therefore, miR-106a is a promising candidate for tumor diagnosis, detection, and prognostic evaluation, as well as for enhancing cancer therapy outcomes^35^.

The microRNA-200 family, which comprises miR-200a, miR-200b, and miR-200c, has been linked to various elements of cancer biology, including epithelial-to-mesenchymal transition (EMT), angiogenesis, chemotherapy resistance, and controls the expression of several significant target genes^36^. In glioma, miR-200a acts as a tumor suppressor *by targeting* the single-minded homolog 2-short form (SIM2-s)^37^, and its downregulation causes glioma proliferation and progression as well as involvement in the therapeutic response^38^. In normal murine mammary epithelial cells, overexpression of miR-200a suppresses EMT by targeting and downregulating ZEB1 and ZEB2 directly through miR-200a-binding sites located on their 3′UTRs^39,40^. The growing importance of miRNAs in biological processes and their potential as diagnostic and prognostic biomarkers have led to the development of numerous experimental and computational approaches for identifying novel miRNA-disease relationships. These methods not only facilitate a deeper understanding of disease pathogenesis but also accelerate the development of molecular tools for diagnosis, treatment, and prevention^41,42^. Hence the current study aimed to investigate the impact of miR-29a, miR-106a, and miR-200a as minimally invasive molecular markers for the prediction and prognosis of GBM patients.

## Results

The current study enrolled forty-five participants: 20 healthy volunteers and 25 newly diagnosed GBM patients. All participants were of matched ages, as no significant differences were observed among them. Regarding gender, twelve patients were females (26.7%), three were healthy volunteers, and the remaining patients were diagnosed with GBM. Thirty-three (51.5%) participants were males, 17 were controls, and 16 (48.5%) were GBM patients. Both demographic and clinicopathological parameters are reported in Table 1.

**Table 1:** Demographic and clinicopathological parameters for GBM cases and control group.

Factors	GBM cases (*n* = 25) *N* (%)	Control group (*n* = 20) *N* (%)
Age		
< 60	13 (52)	17 (85)
≥ 60	12 (48)	3 (15)
Genger		
Male	16 (64)	17 (85)
Female	9 (36)	3 (15)
Tumour size		
< 5 mm	12(48)	
≥ 5 mm	13(52)	
Surgery type		
Excision	11 (44)	
Biopsy	14 (56)	
Primary lesion site		
Non- frontal	12(48)	
Frontal	13(52)	
ECOG		
< 2	13 (52)	
= 2	12 (48)	
Response		
Partial (PR) & Complete (CR) response	6 (24)	
Stable disease	9 (36)	
Progressive disease	10 (4)	

*p value < 0.05: significant.

### MiRs expression levels among the enrolled participants

**Fig. 1:**
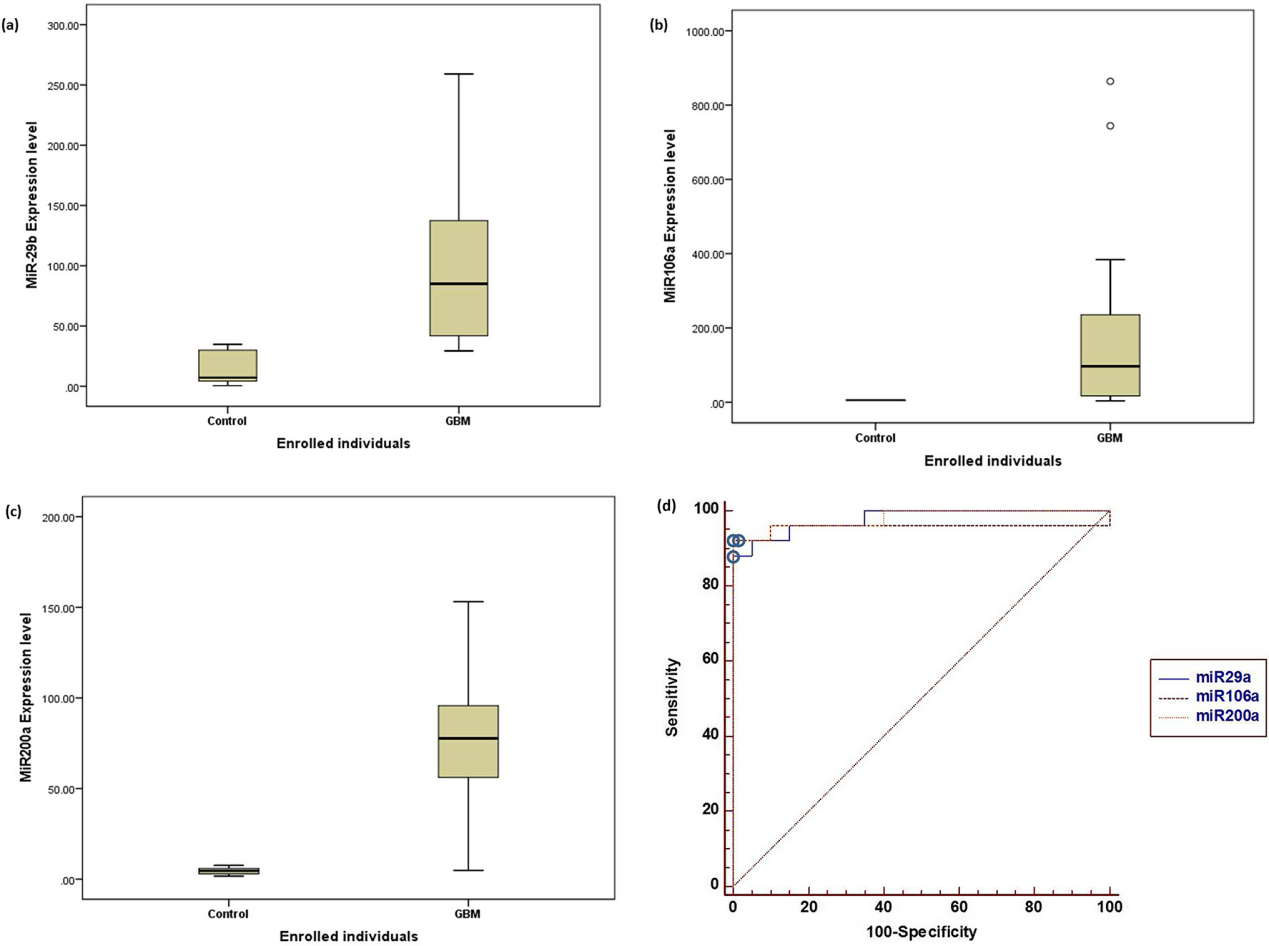
MiRs expression levels among the enrolled participants. (**a**) The mean miR-29a expression level was 14.99 ± 3 for control participants and 106.74 ± 15.26 for GBM patients (F = 28.14, *p* < 0.0001). (**b**) The mean miR-106a expression level was 5.78 ± 0.15 for control participants and 168.43 ± 44.49 for GBM patients (F = 10.64, *p* = 0.002). (**c**) The mean miR-200a expression level was 4.44 ± 0.39 for control participants and 77.24 ± 8.18 for GBM patients (F = 62.99, *p* < 0.0001). (**d**) Receiver operating characteristic (ROC) curves for the investigated miRs. The open circles denote the best cutoff points for discriminating between GBM patients and healthy controls for each investigated miRNA which was 34.78 with 0.978 AUC & CI 95% [0.882–0.996] at (*p* < 0.0001) for miR-29a, and 6.85 with 0.956 AUC & CI 95% [0.849–0.993] at (*p* < 0.0001) for miR-106a, and 7.67 with an AUC of 0.98 & CI 95% [0.885–0.996] at (*p* < 0.0001) for miR-200a.

As shown in Fig. 1a-c, marked significant differences were observed in the expression levels of the investigated miRs in GBM patients, compared to healthy individuals. GBM patients showed highly considerable up-regulation of serum hsa-miR-29a expression compared to the healthy volunteers (mean ± SE = 106.74 ± 15.26-fold change vs. 14.99 ± 3-fold change, F = 28.14, *p* < 0.0001) Fig. 1a. In addition, Fig. 1b revealed marked significant up-regulation in serum hsa-miR-106a expression level in the GBM patient group, compared to the control group (mean ± SE = 168.43 ± 44.49-fold change vs. 5.78 ± 0.15-fold change, F = 10.64, *p* = 0.002). Similarly, the expression level of serum hsa-miR-200a was statistically up-regulated in GBM patients, compared to control participants (mean ± SE = 77.24 ± 8.18-fold change vs. 4.44 ± 0.39-fold-change, F = 62.99, *p* < 0.0001). To investigate the sensitivities and specificities of the investigated miRs, ROC curves were generated (Fig. 1d), which revealed the following sensitivities and specificities: miR-29a: 88% and 100% with an AUC of 0.978 (CI 95% was [0.882-0.996] with an SE ± 0.0217) at *p *< 0.0001; miR-106a: 92% and 100% with an AUC of 0.956 (CI 95% was [0.849-0.993] with an SE ± 0.0308) at *p *< 0.0001; and miR-200a: 92% and 100% with an AUC of 0.980 (CI 95% was [0.885-0.996] with an SE ± 0.0206) at *p *< 0.0001.

### Mean values of the assessed miRs according to clinical criteria

As shown in Table 2, miR-29a was significantly overexpressed in patients with non-frontal lesions and those who underwent biopsy resection. Similarly, the mean miR-200a level was significantly higher in GBM patients ≥ 60 years of age, patients with a tumor size greater than 5 mm, and patients with non-frontal lesions. MiR-106a was elevated significantly in GBM patients with non-frontal lesions, a tumor size > 5 mm, patients who underwent biopsy excision, and patients with an ECOG score of 2.

**Table 2:** MiRs expression levels (mean ± SE) in relation to clinical and GBM response criteria among GBM patients.

Patients’ ages & clinical criteria	miR-29a	miR-106a	miR-200a
Age (years)			
< 60	87.16 ± 18.55	115.49 ± 57.89	57.6 ± 11.25
≥ 60	127.96 ± 23.95	225.78 ± 66.78	98.52 ± 8.67
Statistic	F = 1.85, *p* = 0.187	F = 1.57, *p* = 0.223	F = 8.091, *p* = 0.009*
Gender			
Male	110.67 ± 18.72	199.59 ± 65.5	69.91 ± 8.44
Female	99.75 ± 27.63	113 ± 39.86	90.27 ± 16.93
Statistic	F = 0.114, *p* = 0.739	F = 0.868, *p* = 0.331	F = 1.45, *p* = 0.24
Tumour size			
< 5 mm	83.11 ± 18.17	47.4 ± 21.8	57.38 ± 11.38
≥ 5 mm	128.56 ± 23.1	280.14 ± 71	95.58 ± 9.45
Statistic	F = 2.33, *p* = 0.140	F = 9.15, *p* = 0.006*	F = 6.74, *p* = 0.016*
Surgery type			
Excision	64.55 ± 12.44	49.47 ± 27.54	67.33 ± 14.44
Biopsy	139.9 ± 21.96	261.89 ± 67.37	85.03 ± 9.15
Statistic	F = 7.68, *p* = 0.011*	f- 7.03, *p* = 0.014*	F = 1.162, *p* = 0.292
Site of primary lesion			
Frontal	65.88 ± 11.27	55.72 ± 24.06	61.15 ± 11.33
Non- frontal	151 ± 23.79	290.53 ± 75.53	94.67 ± 9.96
Statistic	F = 11, *p* = 0.003*	F = 9.38, *p* = 0.006*	F = 4.87, *p* = 0.038*
ECOG			
< 2	80.5 ± 19.7	49.72 ± 20	72.6 ± 13.8
= 2	130.97 ± 21.58	278 ± 72.03	81.53 ± 9.63
Statistic	F = 2.95, *p* = 0.099	F = 8.67, *p* = 0.007*	F = 0.289, *p* = 0.596
GBM Response			
CR + PR	69.7 ± 14.75	16.23 ± 3.03	42.07 ± 14.89
SD	58.66 ± 12.18	71.85 ± 33.86	84.02 ± 14.4
PD	172.25 ± 23	346.66 ± 79.17	92.24 ± 9.7
Statistics	F = 11.67, *p* < 0.0001*	F = 9.19, *p* = 0.001*	F = 3.7, *p* = 0.041*

*CR* complete response, *PR* partial response, *PD* progressive disease, *SD* stable disease.

*p value <0.05: significant.

### Relation between miRs expression before and after treatment

**Fig. 2:**
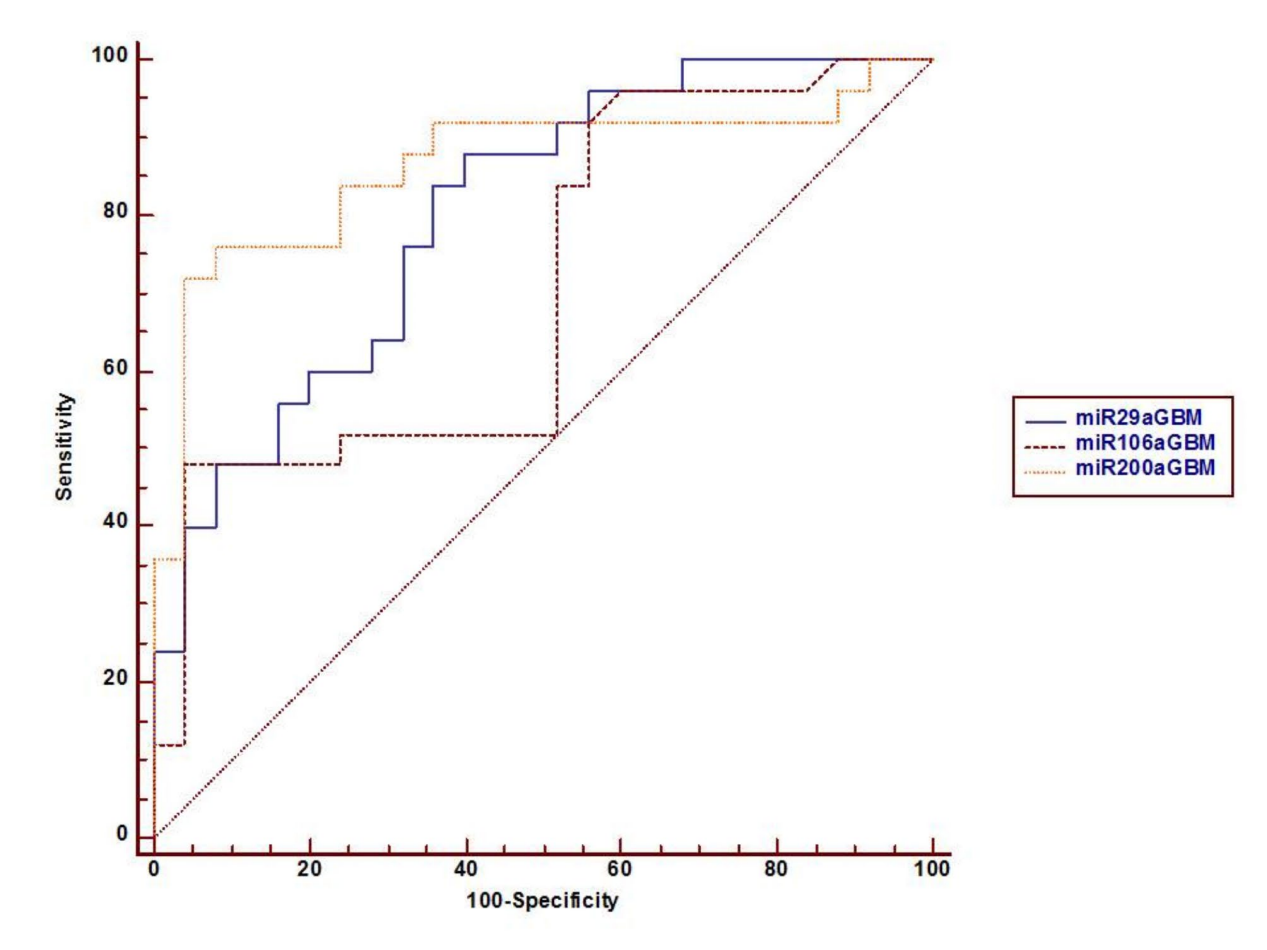
ROC curve representing the expression of the investigated miRs before and after treatment: miR-29a, AUC = 0.802, 95% CI [0.665–0.901], and SE ± 0.0631 at *p* = 0.0001); miR-106a, AUC = 0.707, 95% CI [0.561 to 0.827], and SE ± 0.0737 at *p* = 0.0049); miR-200a, AUC = 0.864 (95% CI [0.737–0.944], with SE ± 0.0531 at *p* = 0.0001).

The expression levels of the assessed miRs were compared before and after treatment among GBM patients, and the results showed that the mean ± SE for miR-29a was 106.74 ± 15.26 vs. 45 ± 6.83 at F = 13.6, *p* = 0.001; for miR-106a, it was 168.8 ± 44.43 vs. 85.57 ± 14.63 at F = 5.55, *p* = 0.023; and for miR-200a, it was 77.24 ± 8.17 vs. 23.38 ± 4.23 at F = 34.2, *p* < 0.0001. Additionally, their levels were compared using ROC curve analysis (Fig. 2) that showed miR-200a had the highest AUC of 0.864 (95% CI [0.737–0.944], with SE ± 0.0531 at *p* = 0.0001), followed by miR-29a, with an AUC of 0.802 (95% CI [0.665–0.901], with SE ± 0.0631 at *p* = 0.0001), and then miR-106a, with an AUC of 0.707 (95% CI [0.561 to 0.827], with SE ± 0.0737 at *p* = 0.0049).

### Effect of miRs expression on the response to treatment

The response to treatment was categorized into CR and PR as a group, SD as a second group and PD as a third group. The levels of the analyzed miRs were reported among these groups and revealed significant differences, as reported in Table 3.

**Table 3:** Level of miRs expression according to GBM response criteria.

	miR29a	miR-106a	miR-200a
GBM response			
CR + PR	69.7 ± 14.75	16.23 ± 3.03	42.07 ± 14.89
SD	58.66 ± 12.18	71.85 ± 33.86	84.02 ± 14.4
PD	172.25 ± 23	346.66 ± 79.17	92.24 ± 9.7
Statistics	F = 11.67, *p* < 0.0001	F = 9.19, *p* = 0.001	F = 3.7, *p* = 0.041

*CR* complete response, *PR* partial response, *SD* stable disease, *PD* progressive disease.

### Predictive power of investigated miRs among survival patterns

**Fig. 3:**
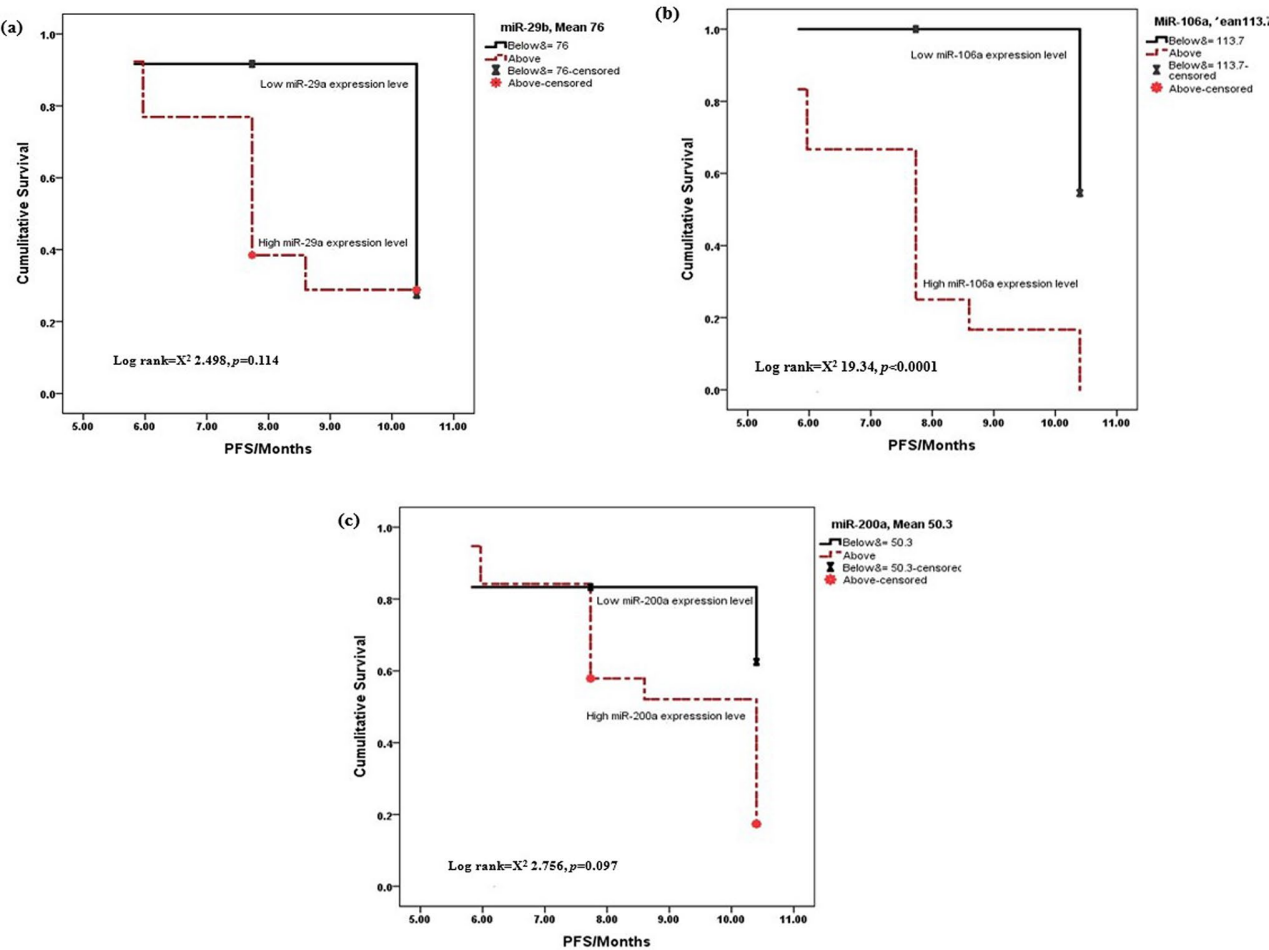
Kaplan–Meier curves showing the relationship between the investigated miRs and progression-free survival in GBM patients. (**a**) miR-29a, mean ≤ 76 (95% IC [9.256–10.783]) and mean>76 (95%IC [7.266–9.070]), *p* = 0.114, (**b**) miR-106a, mean ≤ 113.7 (95% IC [10.400–10.400]) and mean>113.7 (95%IC [6.731–8.547]), *p* < 0.0001 and (**c**) miR-200a, mean ≤ 50.3 (95% IC [7.713–11.565]) and mean>50.3 (95%IC [8.087–9687]), *p* = 0.097.

**Fig. 4:**
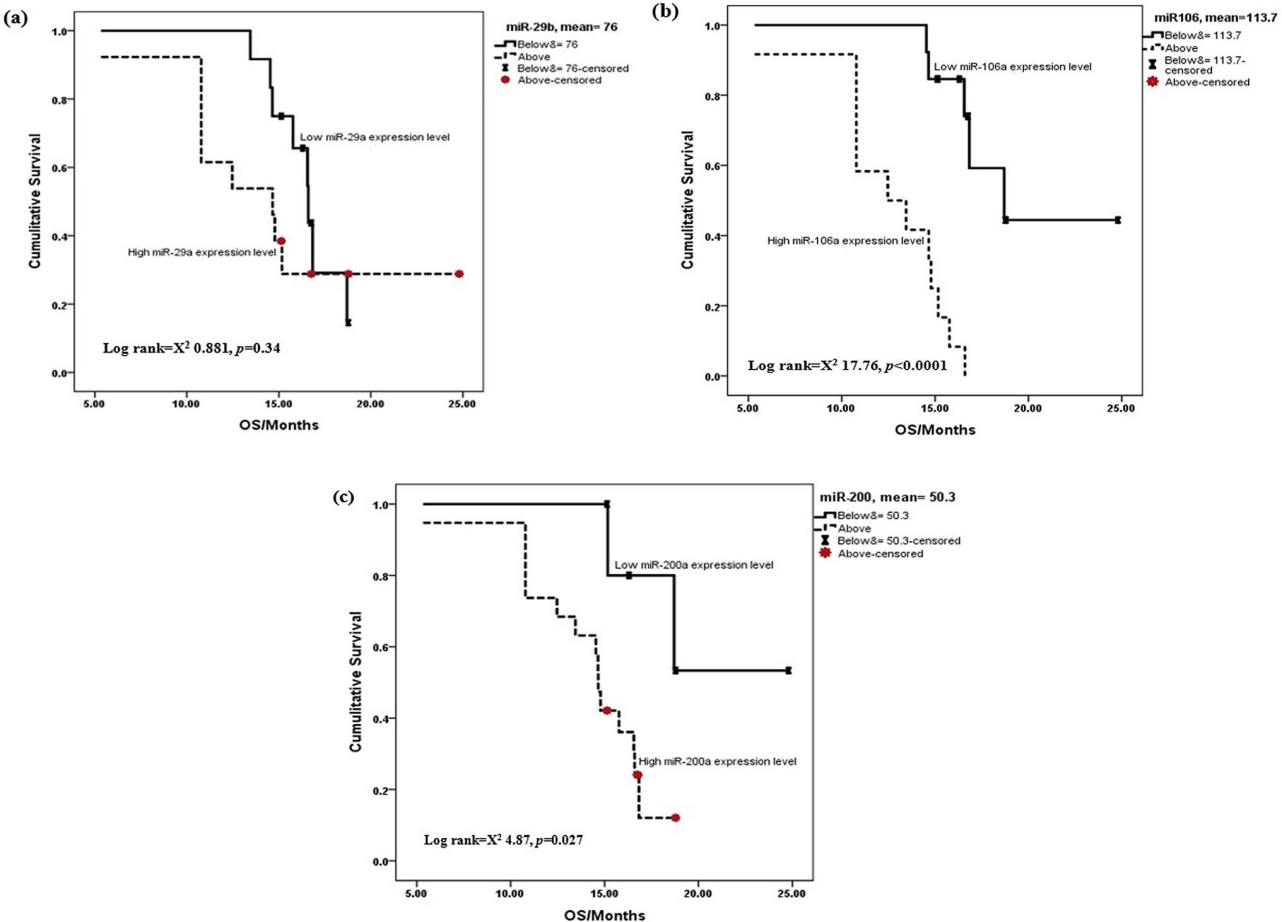
Kaplan–Meier curves showing the relationship between the investigated miRs and overall survival (OS) in GBM patients. (**a**) miR-29a, mean ≤ 76 (95% IC [15.548–17.608) and mean>76 (95%IC [12.040–19.097]), *p* = 0.384, (**b**) miR-106a, mean ≤ 113.7 (95% IC [17.447–23.102]) and mean>113.7 (95%IC [10.849–14.386]), *p* < 0.0001 and (**c**) miR-200a, mean ≤ 50.3 (95% IC [17.521–24.972]) and mean>50.3 (95%IC [12.719–15.741]), *p* = 0.027.

The mean levels of the assessed miRs were 76-, 113.7-, and 50.3-fold higher for miR-29a, miR-106a, and miR-200a, respectively, and GBM patients were grouped as above or below the mean level. Accordingly, GBM patients with low mean expression levels of miR-106a were reported to have better PFS, as shown in Fig. 3a–c, and a significant difference was detected between the two groups. Regarding overall survival (OS), patients with low expression of the investigated miRs had better OS, and the expression levels of both miR-106a and miR-200a were significantly higher, as shown in Fig. 4a-c.

### Functional enrichment analysis

**Fig. 5:**
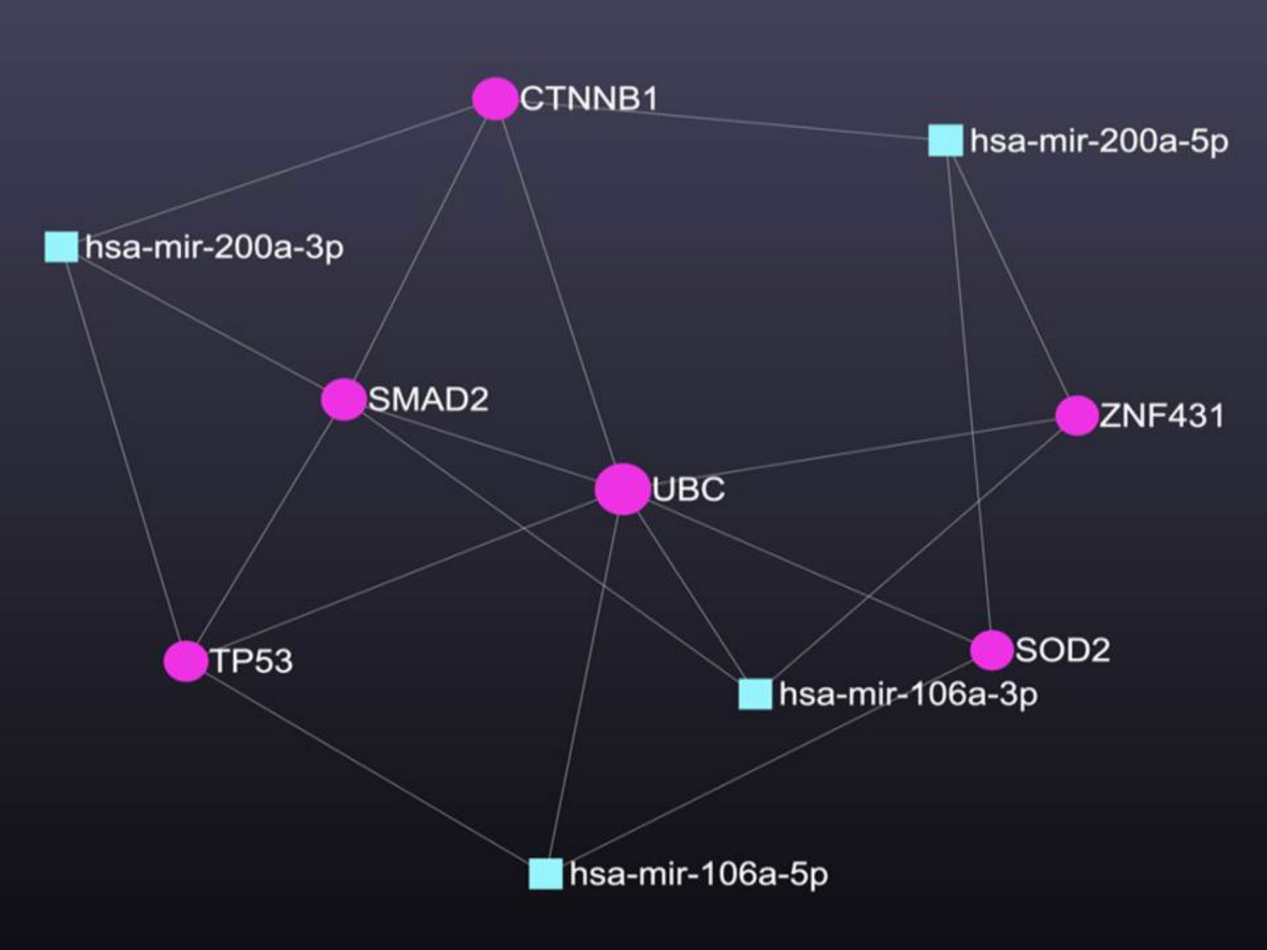
Network enrichment analysis showing the studied miRNAs and their direct protein targets.

MiR-106a and miR-200a were enriched against GBM as a disease term on the miRNet2 web server, and the resulting network was minimized using the minimum network option. The resulting network represented miRs interacting with the targeted proteins; hsa-miR-2001-3p was found to directly regulate 3 important proteins, TP53, SMAD2, and CTNNB1, while hsa-miR-200a-5p was found to regulate CTNNB1, ZNF431, and SOD. hsa-miR-106-3p was found to regulate SMAD2, UBC, and ZNF431, and hsa-miR-106a-5p regulated TP53, UBC, and SOD2 as shown in Fig. 5.

**Fig. 6:**
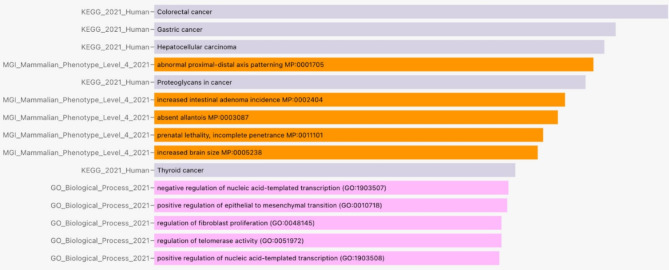
KEGG enrichment analysis of the 3 main players in the cancer pathway (https://www.kegg.jp/kegg/kegg1.html).

Network enrichment analysis showing the studied miRNAs and their direct protein targets. Pathway enrichment analysis was carried out, and the resulting proteins from the network enrichment were enriched using KEGG, which is available on the miRNet2 web server https://www.mirnet.ca/miRNet, to elucidate the common pathway that includes those genes. As shown in Fig. 6, the results revealed that the main enriched pathways were involved in cancer, the adherent junction pathway, and the Wnt signaling pathway, which are the three main players CTNNB1, SMAD2, and TP53. The subnetwork shows the following associations: From Gene Ontology: SMAD2, and CTNNB1 belong to the biological process of positive regulation of epithelial to mesenchymal transition (GO:0010718). CTNNB1 and TP53 belong to the biological process regulation of fibroblast proliferation (GO:0048145). SMAD2, CTNNB1, and TP53 belong to the biological process of positive regulation of nucleic acid-templated transcription (GO:1903508). SMAD2, CTNNB1, and TP53 belong to the biological process of negative regulation of nucleic acid-templated transcription (GO:1903507). CTNNB1 and TP53 belong to the biological process regulation of telomerase activity (GO:0051972). From MGI Mammalian Phenotype: The phenotype prenatal lethality, incomplete penetrance MP:0011101 was observed in SMAD2, CTNNB1, and TP53 KO mice. The phenotype increased brain size MP:0005238 was observed in CTNNB1, and TP53 KO mice. The phenotype absent allantois MP:0003087 was observed in SMAD2 and CTNNB1 KO mice. The phenotype of abnormal proximal–distal axis patterning MP:0001705 was observed in SMAD2 and CTNNB1 KO mice. The phenotype increased intestinal adenoma incidence MP:0002404 was observed in CTNNB1 and TP53 KO mice. From KEGG: The gene products SMAD2, CTNNB1, and TP53 are members of the KEGG pathway Hepatocellular carcinoma. The gene products SMAD2, CTNNB1, and TP53 are members of the KEGG pathway of gastric cancer. The gene products SMAD2, CTNNB1, and TP53 are members of the KEGG pathway for colorectal cancer. The gene products CTNNB1 and TP53 are members of the KEGG pathway Thyroid cancer. The gene products SMAD2, CTNNB1, and TP53 are members of the KEGG pathway Proteoglycans in cancer.

## Discussion

Dysregulation of miRNAs is one of the main mechanisms involved in GBM development^43^. Identification of novel sensitive and more specific molecular markers is urgently needed to develop precise medical approaches for disease management. Circulating miRNAs represent prospective biomolecules in cancer management since they are easily detectable and highly stable in biofluids, providing non-invasive methods that may be repeated over time without causing pain or stress for cancer patients. Therefore, it is plausible that miRNAs might be incorporated into the current prognostic framework to improve patient management. Exhaustive studies revealed the usefulness of miRs as molecular indicators for prompt diagnosis and prognostic assessment for several types of cancers, including GBM^14,18,44,45^.

The current study aimed to assess the predictive and prognostic value of specific circulating miRs in patients with GBM. Earlier studies have documented the function of miRs as oncomiRs and tumor suppressors in cancer development^46,47^. Our results revealed highly significant differences in the expression levels of miR-29a, miR-106a, and miR-200a in GBM patients compared to healthy volunteers, indicating that the identified miRs could be potential molecular markers for GBM diagnosis and prediction. These findings are consistent with those of Shi and his colleagues^22^, who reported that miR-29a, miR-29b, and miR-29c could be attractive prognostic markers of brain tumors. Additionally, Du and his colleagues^48^ revealed the upregulation of miR-106a in acute cerebral infarction (ACI) patients, indicating its significance as a novel minimally invasive diagnostic and prognostic biomarker.

The mean expression levels of the investigated miRs were assessed considering the clinical criteria of GBM patients. Mei and her colleagues^49^ reported that miR-29a expression increases with age and is involved in neuronal maturation and the inflammatory response. In line with this concept, our data revealed that miR-29a and miR-106a were overexpressed in patients older than 60 years, but these increases were not significant, which may be due to the small sample size reported in the present study. A significant difference in the miR-200a expression level was observed among patients older than 60 years. These results may indicate the distinctive dysregulated molecular profile of elderly GBM patients^50^. No significant difference was reported between the investigated miRs and sex, which was consistent with the findings of a previous study by Mooney and his colleagues^51^.

The invasive behavior of GBM cells is the main factor for poor patient prognosis^52^. During invasion, cancer cells gain the ability to destroy the extracellular matrix and then penetrate nearby normal tissue; this process can be inhibited by modifying effector molecules that can significantly enhance patient prognosis^53^. The results of the present study showed that significant overexpression of miR-106a and miR-200a was associated with a tumor size ≥ 5 mm in GBM patients. GBM patients were categorized into two groups according to the site of the primary lesion: those with tumors at the frontal site (*n* = 13) and those with tumors at non-frontal sites (*n* = 12). A significant increase in the expression levels of the investigated miRs was detected among the non-frontal site lesions. These findings indicate the oncogenic properties of this type of cancer and the aggressiveness of these tumors.

Circulating miRs may have prognostic and predictive value for improving patient selection for sedative treatment^21,27,43^. In the present study, the three detected miRs were significantly expressed at the tumor site and may have prognostic value. Additionally, the downregulation of their expression level that was observed among GBM patients with frontal site lesions rendered them prone to full surgical amputation^54^.

Moreover, relative to the ECOG score, a helpful indicator of tumor performance^55^, the current results revealed upregulation of the expression of the investigated miRs, highlighting the potential importance of these liquid biopsy samples as predictors for a better prognosis. Fluidic biopsy provides a desirable minimally invasive option to boost various stages of GBM management, evaluate tumor molecular markers, detect longitudinal genomic evolution, anticipate patient prognosis, predict prospective resistance to radiotherapy and chemotherapy, and permit patient selection for reliable therapies^4^.

With advances in molecular technologies, abnormal miRNA expression was revealed to be a remarkable factor affecting the sensitivity of cancer cells to therapy. As either oncomiRs or tumor suppressors, miRNAs may be involved in the initiation and progression of cancer cell resistance to chemotherapy^29^. After treatment, the present study demonstrated significant downregulation of the expression levels of the investigated miRs. These results may indicate the potential participation of miRs as novel predictors of response to therapy and the prospective prognosis of GBM patients.

Present results are consistent with those of previous studies^43,56^, as they suggested miR-200a and miR-106a could be used to predict prognosis and represent promising new therapeutic targets for GBM patients. Moreover, Chen et al.^57^ reported that the expression level of miR-29a was inversely correlated with the tumor grade of human GBM; in vitro, miR-29a can suppress glioma cell proliferation, migration, and invasion while inducing apoptosis. P53 upregulates the expression level of miR-29a, causing abnormal expression of its target MDM2 and eventually unbalancing p53-miR-29a-MDM2 feedback-loop activity. Furthermore, miR-29a controls p53/MDM2 signaling, increasing the responsiveness of glioma cells to TMZ. Additionally, Xu et al.^58^ demonstrated that miR-29b enhances TMZ sensitivity in GBM cells by activating autophagy and that combining a miR-29 mimic with TMZ may constitute an alternative therapeutic approach for GBM patients.

Following the standard therapy for GBM, patients underwent additional monitoring, and their MR images were reanalyzed to improve consistency and reduce intraobserver variability. Consequently, significant differences were observed in the expression levels of miRs, where miR-106a showed a statistically significant reduction in expression levels in patients with better PFS and OS. These findings support the critical clinical function of miR-106a in predicting the survival of individuals with GBM. This result is consistent with those of previous studies by Zhao et al.^30^, who suggested that miR-106a expression was a significant and autonomous predictor of OS in GBM patients and that miR-106a downregulation was significantly correlated with the progression of malignancy. Regarding miR-200a, the present study revealed a significant reduction among patients with better OS.

In conclusion, the present study uniquely pointed to the specific roles of miR-29a, miR-106a, and miR-200a concerning GBM diagnosis and prognosis among Egyptian patients. It focused on the role of three miRs—miR-29a, miR-106a, and miR-200a—as stable liquid biopsy markers and emphasized their significant role in discriminating between GBM patients and healthy controls. The study also revealed the usefulness of these miRNAs for predicting patient response to treatment and their role as prognostic markers that can predict survival patterns (PFS and OS) in GBM patients. MiRNAs analysis using advanced molecular techniques (qRT-PCR) in combination with clinical data and in silico analysis offers valuable insights into GBM diagnosis, prognosis, and treatment outcomes providing valuable insights for personalized therapeutic strategies. These miRNAs have been linked to critical cancer pathways like Wnt signaling and TP53 regulation, but their distinct association with non-frontal lesions, age-related tumor size, and surgical strategy outcomes make this study particularly relevant for exploring their predictive value. The study also identified miR-200a as having a significant correlation with tumor size in patients over 60 years old and miR-106a with different surgical strategies. Furthermore, a significant downregulation of all target miRNAs post-treatment, suggests these miRNAs could serve as markers to monitor treatment efficacy over time.

Although the current study reports the importance of using miRNAs as liquid biopsy markers for detection and the prediction of patient response to treatment and as prognostic markers, the number of samples recruited in the study is small, which stresses the importance of enrolling more GBM patients to confirm the current findings. The study is in progress to accomplish this assignment. In addition, future studies are ongoing to detect the expression of miRNAs extracted from exosomes and compare their levels with those extracted from serum to compare their sensitivities as liquid biopsy markers for better diagnosis, prediction, and prognosis of GBM.

## Materials and methods

### Ethical approval and enrolled individuals

All enrolled participants in this study provided written informed consent after receiving clearance from the Research Ethical Committee (REC), Faculty of Medicine, Ain Shams University (Approval No. FWA 000017585). This research was conducted on newly diagnosed GBM patients (*n* = 25:9 females and 16 males) admitted to the Clinical Oncology Department, Faculty of Medicine, Ain Shams University, Cairo, Egypt. In addition, a group of healthy volunteers (*n* = 20) were enrolled as reference controls. The inclusion criteria for GBM patients were adults (aged > 18 years) who were newly diagnosed with GBM with a performance score less than 2 (matching with Eastern Cooperative Oncology Group [ECGO])^59,60^, and before they received any treatment modalities, any other patients who did not fulfill the inclusion criteria were excluded from the study. The healthy volunteers were enrolled from among laboratory staff members after assuring that they were not suffering from any type of malignancy or other disease (inflammation or degenerative disease) that could affect the level of the investigated miRNAs.

### Treatment modalities

Recruited GBM patients were evaluated clinically (through complete history, clinical and neurologic examination) and with brain imaging (magnetic resonance imaging [MRI]) to receive their standardized treatment protocol as previously reported^61^, which included maximum safe resection (if possible), followed by radiotherapy conventional fractions (total dose of 60 Gy, given 2 Gy per fraction for 30 fractions over 6 weeks) or hypofractionation (45 Gy in 15 fractions over 3 weeks) with concomitant temozolomide (TMZ) chemotherapy (75 mg/m2 every day until the end of radiotherapy) with regular follow-up, then re-evaluated clinically followed by adjuvant followed by six cycles of TMZ treatment at a dose of 150 mg/m^2^ body surface area from days 1 to 5 every 28 days with clinical monitoring, except if there was clinical deterioration necessitating radiological assessment. During regular clinical follow-up, patients were assessed by gadolinium-enhanced MRI (Gd-MRI) 45 days after RT and then every 3 months or at the time of clinical evidence of neurologic progression. Tumor response was evaluated based on the radiological RANO response criteria (2010)^62^ of a previous report by Stupp et al.^63^. Complete response (CR): disappearance of all known brain lesions. Partial response (PR) was defined as a 50% or greater decrease in measurable brain lesions or an objective improvement in evaluable brain lesions. Stable disease (SD): brain lesion unchanged (< 50% decrease or < 25% increase in the size of measurable lesions). Progressive disease (PD): ≥ 25% increase in the size of some or all brain lesions and/or the appearance of any new brain lesions.

### Blood processing and storage

Blood samples (3 ml) were collected into serum separator vacutainers (Greiner Bio-One, GmbH, Wetherill Park, Australia). After clotting at room temperature (RT) for 30 min, the samples were centrifuged at 10,000 × g for 10 min at 4 °C. Aliquots of separated serum samples were stored at -80°C for miR analysis.

### Isolation of miRs, reverse transcription and synthesis of complementary DNA (cDNA), and quantitative real-time PCR (qRT‒PCR)

The method for miRs analysis was described in detail in a previous study^15^. According to the manufacturer’s instructions, the miRNeasy Mini kit (Catalog # 217004, Qiagen, USA) was used to extract circulating miRs from the participants’ serum. Then the concentration and purity of the isolated RNA were determined using a Nanodrop spectrophotometer (Quawell, Q-500, Scribner, USA). A MiScript II reverse transcription kit (Cat number # 218160, Qiagen, USA) was used to synthesize cDNA from the miRs. Then, a miScript SYBR Green PCR kit (# 218073, Qiagen, USA) was used to quantify the investigated miRNAs according to the manufacturer’s protocol. The primers used were miR-29a (Hs_miR_29a_1 miScript Primer Assay, MS00003262), miR-106a (Hs_miR_106a_1 miScript Primer Assay, MS00008393), and miR-200a (Hs_miR_200a_1 miScript Primer Assay, MS00003738). The endogenous control used was RNU6-2 (Hs RNU6-2_11 miScript Primer Assay: MS00033740). A qRT-PCR system (Max3005P qPCR System, Stratagene, Agilent Biotechnology, USA) was utilized to detect the acquired fluorescence. Fold changes of the investigated miRs were calculated using the ΔCt method. ΔCt indicates the disparities in the cycle threshold numbers between the assessed miRs and the endogenous control. The ΔΔCt value represents the difference between the studied miRs in GBM patients and healthy volunteers^64^.

### In silico analysis

Network enrichment analysis was performed using the miRNet2 online server to enrich miR-106a and miR-200a against glioblastoma functionally, and the resulting protein–protein interaction list was then used for pathway enrichment analysis via the Enrichr web-based tool^65–67^ (https://maayanlab.cloud/Enrichr/#) and the images were picked up from enricher-KG^68^ (https://maayanlab.cloud/enrichr-kg) through KEGG database^67,69–71^.

### Statistical analysis

**Fig. 7:**
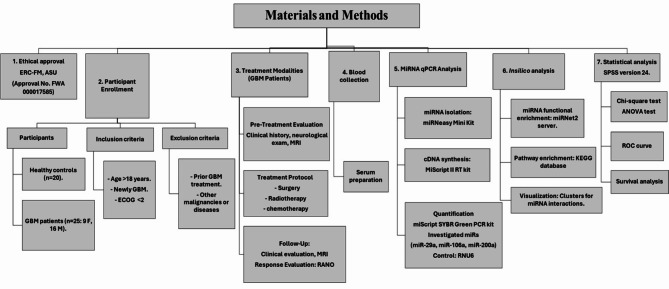
Flowchart represents the materials and the methods for the study experimental design.

Data analysis was performed using SPSS version 24 (SPSS, Inc., Chicago, USA), and *p* values < 0.05 were considered to indicate statistical significance. The 2-^ΔΔCT^ equation was utilized to compute the fold change expression of the investigated miRs. Variations between categorical variables were detected using chi-square tests. ANOVA was used to determine the associations between clinicopathological and demographic parameters and the miRs under investigation. Plotting a receiver operating characteristic (ROC) curve between GBM patients and healthy volunteers allowed researchers to identify the sensitivity, specificity, and therapeutic effectiveness of the miRs under investigation. The duration between the first administration of the neoadjuvant therapy approach and the earliest instance of distal, regional, or local recurrence was defined as progression-free survival (PFS). Overall survival (OS) was computed from the date of the initial diagnosis to the date of the last follow-up or death using the Kaplan‒Meier method and a log-rank test, the materials, and the methods for the study’s experimental design were shown in a flowchart Fig. 7.

## Data Availability

The datasets generated and analyzed during the current study are available from the corresponding author upon reasonable request.
